# Synergy of Physico-chemical and Biological Experiments for Developing a Cyclooxygenase-2 Inhibitor

**DOI:** 10.1038/s41598-018-28408-8

**Published:** 2018-07-03

**Authors:** Palwinder Singh, Jagroop Kaur, Harpreet Kaur, Anudeep Kaur, Rajbir Bhatti

**Affiliations:** 1 Department of Chemistry, Centre for Advanced Studies, Guru Nanak Dev University, Amritsar, 143005 India; 2 Department of Pharmaceutical Sciences, Guru Nanak Dev University, Amritsar, 143005 India

## Abstract

The physiological consequences of COX-2 overexpression in the development of cancer, diabetes and neurodegenerative diseases have made this enzyme a promising therapeutic target. Herein, COX-2 active site was analyzed and new molecules were designed. We identified a highly potent molecule (*S*)-**3a** with IC_50_ value and the selectivity for COX-2 0.6 nM and 1666, respectively. The MTD of (*S*)-**3a** was 2000 mg kg^−1^ and its pharmacokinetic studies in rat showed t_1/2_ 7.5 h. This compound reversed acetic acid induced analgesia and carragennan induced inflammation by 50% and 25% in rat when used at a dose 10 mg kg^−1^. Mechanistically, it was found that compound (*S*)-**3a** inhibits COX-2. Overall, the combination of physico-chemical and biological experiments facilitated the development of a new lead molecule to anti-inflammatory drug.

## Introduction

The role of two isoforms of cyclooxygenase- COX-1 and COX-2 in the synthesis of house-keeping prostaglandins and inflammatory prostaglandins, respectively during the arachidonic acid metabolic pathway is well documented^1–4^. Henceforth, the development of COXIBS such as celecoxib as the anti-inflammatory drug in the late 1999 shifted the focus of non-steroidal anti-inflammatory drugs towards selective COX-2 inhibitors^5–10^. However, the association of ulceration and cardiovascular^11–17^ side effects with these drugs, resulting in the withdrawal of rofecoxib and valdecoxib from the market, has restrained the medical applications of COXIBS. Therefore, the search for new chemical entities with safe and efficient medicinal features is highly desirable.

**Figure 1:**
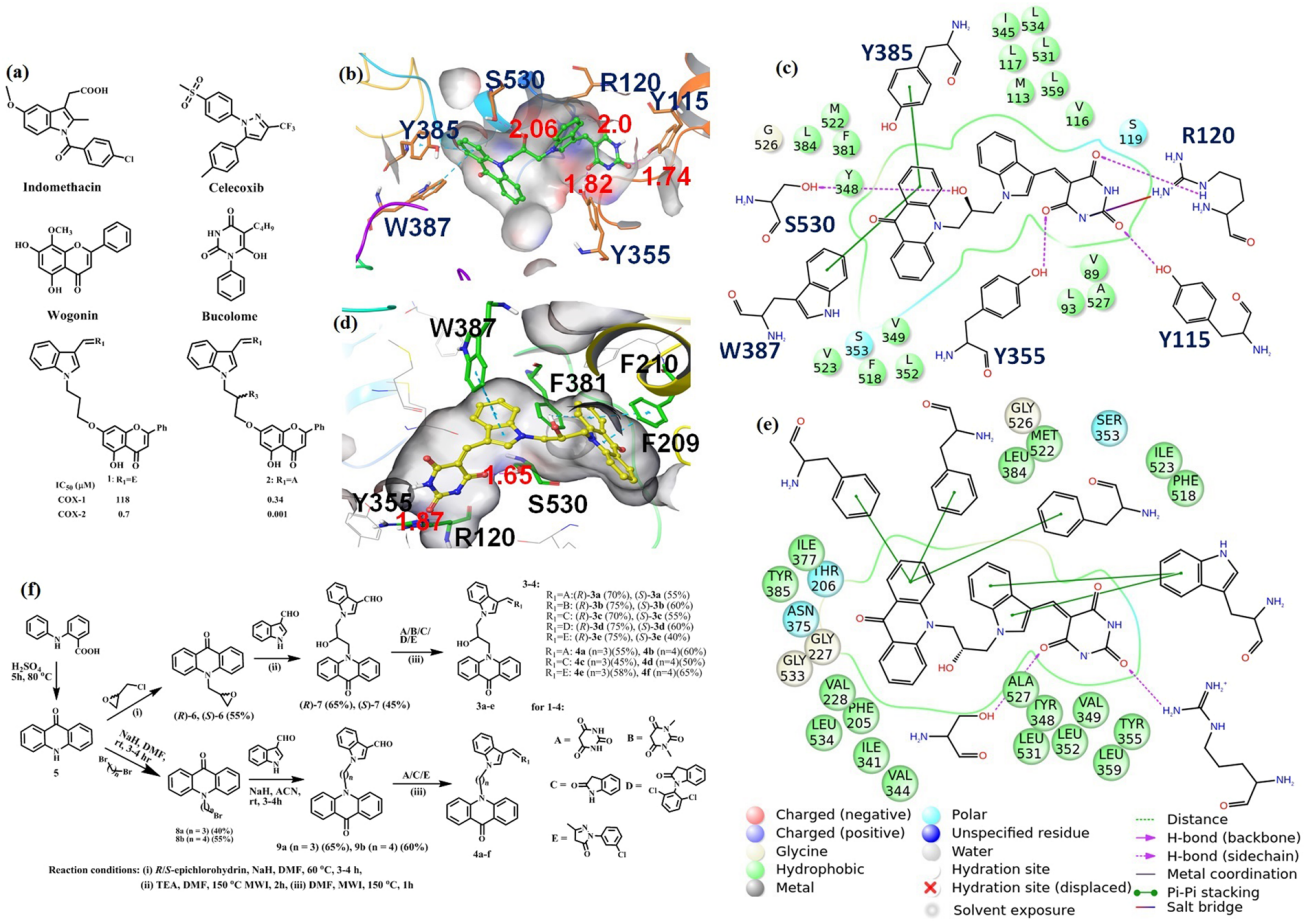
(**a**) Compounds in the clinical use. Compounds **1** and **2** were obtained by the integration of the clinically used drugs. (**b**) Compound (*S*)-**3a** docked in the active site of COX-2 showing H-bond interactions (pink dotted lines, distances in Å) and π-π interactions (blue dotted lines) with active site residues (Hs’ are omitted for clarity). (**c**) 2D view of compound (*S*)-**3a** docked in COX-2. (**d**) Compound (*S*)-**3a** docked in the active site of COX-1 showing H-bond interactions (pink dotted lines, distances in Å) and π-π interactions (blue dotted lines) with active site residues (Hs’ are omitted for clarity). (**e**) 2D view of compound (*S*)-**3a** docked in COX-2. (**f**) Synthesis of compounds **3** and **4**.

No doubt, the availability of the crystal structure of COX-2 – AA complex has assisted in the design of new molecules^18,19^ but the number of candidates which are getting through clinical tests is insignificant^20^. During our recent efforts in combining the template of indomethacin, celecoxib, wogonin, bucolome (Fig. 1a) in one molecule, we were able to identify considerably potent COX-2 inhibitors (**1, 2**; Fig. 1a) that were capable of reducing inflammation in the animal models^21,22^. Since in addition to the polar interactions, hydrophobic interactions play significant role in the inhibition of COX-2 by the anti-inflammatory drugs^23^, it was envisaged that the replacement of the chrysin unit of compound 1 and 2 with acridone moiety may increase its π-π interactions with the enzyme. Hence, new compounds **3** and **4** (Fig. 1f) were designed. The molecular dockings of the compounds in the COX-2 active site were performed (supporting information) and it was observed that the presence of acridone moiety in molecules 3 is engaged in hydrophobic interactions in the COX-2 active site, specifically with Y385, the residue which otherwise actively participates in the electron transfer process during the metabolic phase of the enzyme (Fig. 1b,c). Moreover, the OH group present at the linker between acridone and indole moiety in compound **3a** is engaged in H-bonding with S530, the residue responsible for holding AA during the metabolic phase. The pyrimidine moiety of (*S*)-**3a** showed extensive interactions with R120 present at the entry point of the active site pocket of COX-2. In contrast to above mentioned molecular docking results, compound (*S*)-**3a** exhibited a few interactions with COX-1 (Fig. 1d,e), specifically, the ligand did not interact with Y385 and there was only one interaction with R120. Therefore, the careful investigation of the COX-2 active site and intensive docking studies created the platform for investigating the medicinal properties of compounds **3** and **4**. Compounds **3** and **4** were synthesized and studied for their interactions with COX-2 and were evaluated for COX-2 inhibitory and anti-inflammatory properties.

## Result and Discussion

### Chemistry

As depicted in Fig. 1f, acridone 5 was prepared by the treatment of N-phenyl anthranillic acid with H_2_SO_4_. The reaction of 5 with (*S*)-epichlorohydrin in DMF at 60 °C using NaH as base resulted into the formation of compound (*S*)-6. Similarly, the reaction of **5** with (*R*)-epichlorohydrin provided compound (*R*)-6. Further, the microwave irradiations (MWI) at 150 °C of a mixture of (*R*)-6/(*S*)-6, indole-3-carboxaldehyde and triethyl amine in dimethyl formamide (DMF) led us to procure product (*R*)-**7/**(*S*)-**7**. In order to synthesize compound (*R*)-**3a**/(*S*)-**3a**; an equivalent mixture of (*R*)-**7/**(*S*)-**7** and barbituric acid in DMF was irradiated under MW at 150 °C for 1 h (Fig. 1f). Similarly, the reaction of (*R*)-**7** and (*S*)-**7** with N,N-dimethyl barbituric acid gave compound (*R*)-3b and (*S*)-3b.

Due to the anti-inflammatory potential^24–27^ of oxindole and pyrazole moieties, it was also planned to combine these heterocycles with indole-acridone adduct. Accordingly, compounds 3c–3e were prepared by treating (*R*)-**7** and (*S*)-**7** with oxindole, 1-(2,6-dichlorophenyl)−2-indolinone and 1-(3-chlorophenyl)-3-methyl-2-pyrazolin-5-one, respectively (Fig. 1f).

For the synthesis of compounds 4; first acridin-9-one (5) was treated with dibromopropane and dibromobutane in the presence of NaH in DMF and respectively compounds 8a and 8b were obtained. Compounds 8a and 8b were further reacted with indole-3-carboxaldehyde in acetonitrile (ACN) in the presence of NaH at room temperature for procuring compounds 9a and 9b, respectively. Finally, the MWI of an equivalent mixture of 9a and barbituric acid in DMF at 150 °C for 1 h resulted into the formation of compound 4a. Similarly, 4b was synthesized by treating 9b with barbituric acid. Using the same reaction conditions, compounds 4c and 4d were prepared by reacting 9a and 9b with oxindole whereas compounds 4e and 4f were procured by the reaction of 9a and 9b with 1-(3-chlorophenyl)-3-methyl-2-pyrazolin-5-one (Fig. 1f).

**Figure 2:**
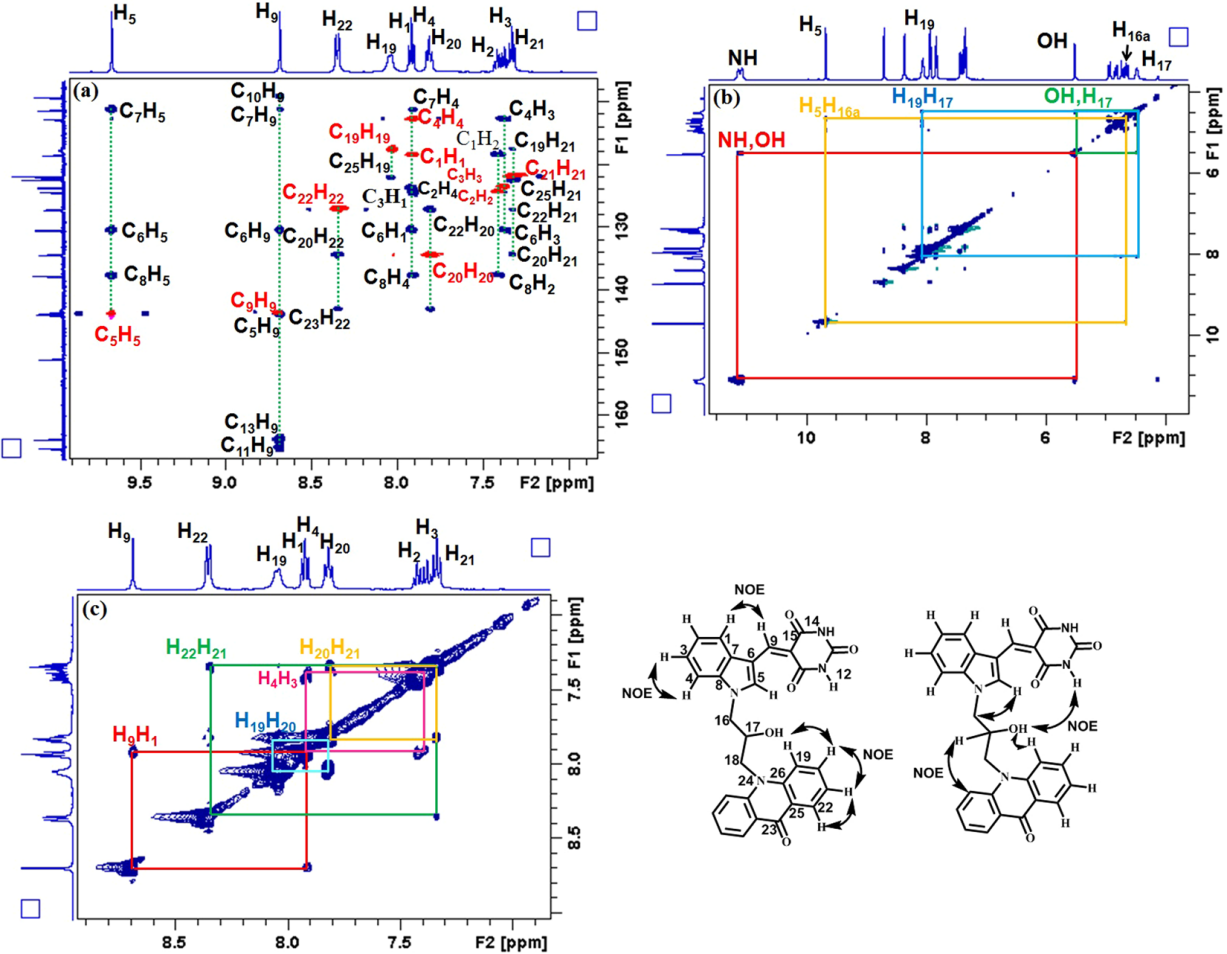
(**a**) An overlay of HSQC (red contours) and HMBC (blue contours) NMR spectra of (*S*)-**3a** for the assignment of H and C chemical shifts. (**b**), (**c**) ^1^H^−1^H NOESY NMR spectrum of compound (*S*)-**3a** confirming *Z*- configuration at bridged C = C.

All the synthesized compounds were characterized by 1D, 2D NMR, IR and HRMS experiments. With the help of nuclear overhauser experiments (NOE) on compound **3a**, *Z*-configuration across the bridged C=C bond was established (Fig. 2, Fig. S67–S77). It was evident from the comparable chemical shift of indoleC-2H (that probably arises due to the H-bond interaction of indoleC-2H with the C=O group) that all the other molecules also have *Z*-configuration at the bridged C=C bond. The percentage purity of the compounds was ascertained with the help of quantitative NMR (q^1^HNMR) spectroscopy by using dimethyl sulphone as the internal calibrant and all the compounds displayed >98% purity (Supporting Information).

### Physico-chemical experiments for interactions of the compound with cyclooxygenase-2 Isothermal Titration Calorimetric (ITC) Studies

**Figure 3:**
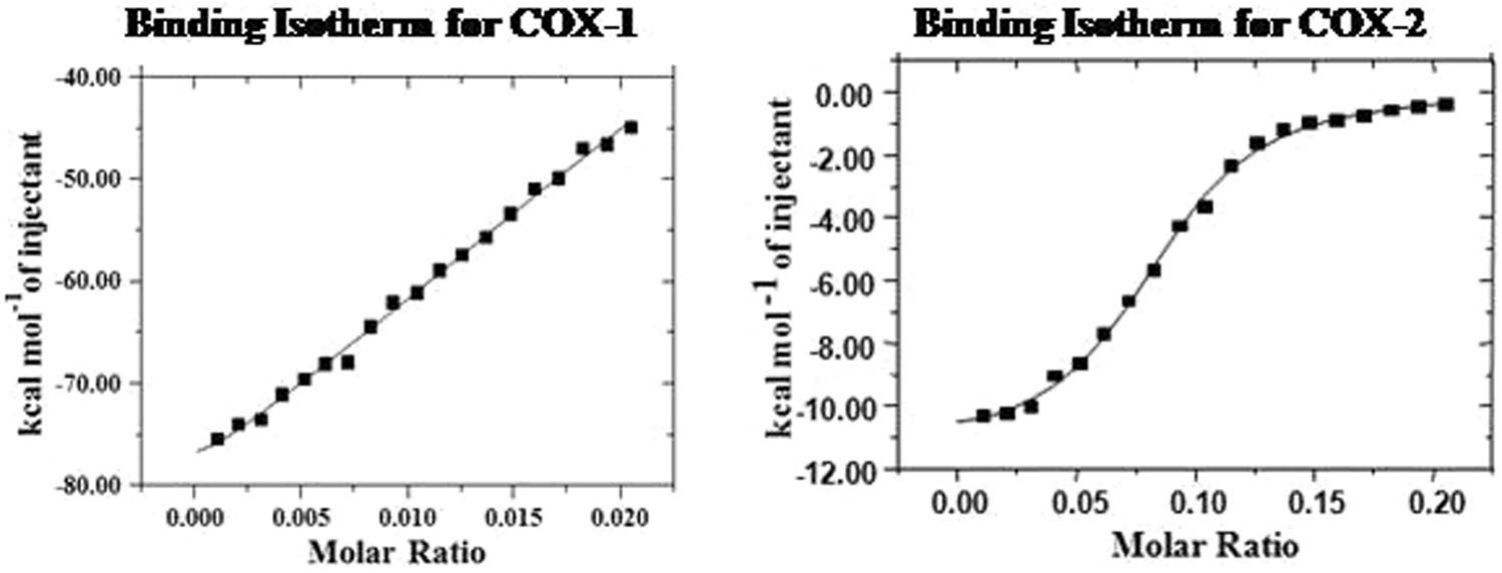
Binding isotherm of compound (*S*)-**3a** for (**a**) COX-1 and (**b**) COX-2.

Association constant, energy and entropy changes for compound (*S*)-**3a** were determined from ITC experiments. It was observed that compound (*S*)-**3a** exhibits highly promising interactions with COX-2 (Table 1, Fig. 3). Subsequently, UV-vis and NMR experiments were performed with compound (*S*)-**3a**.

**Table 1:** Isothermal calorimetric data of compound (*S*)-**3a** for COX-1 and COX-2 enzyme.

Physical parameters	(S)-**3a**	
	COX-1	COX-2
K_a_(M^−1^)	(8.63 ± 0.161) × 10^4^	(1.53 ± 0.117) × 10^6^
ΔH (kJ/mol)	−21.48	−46.59
TΔS (kJ/mol)	−3.47	−11.82
ΔG (kJ/mol)	−18.01	−34.77

### UV-Visible spectral studies

**Figure 4:**
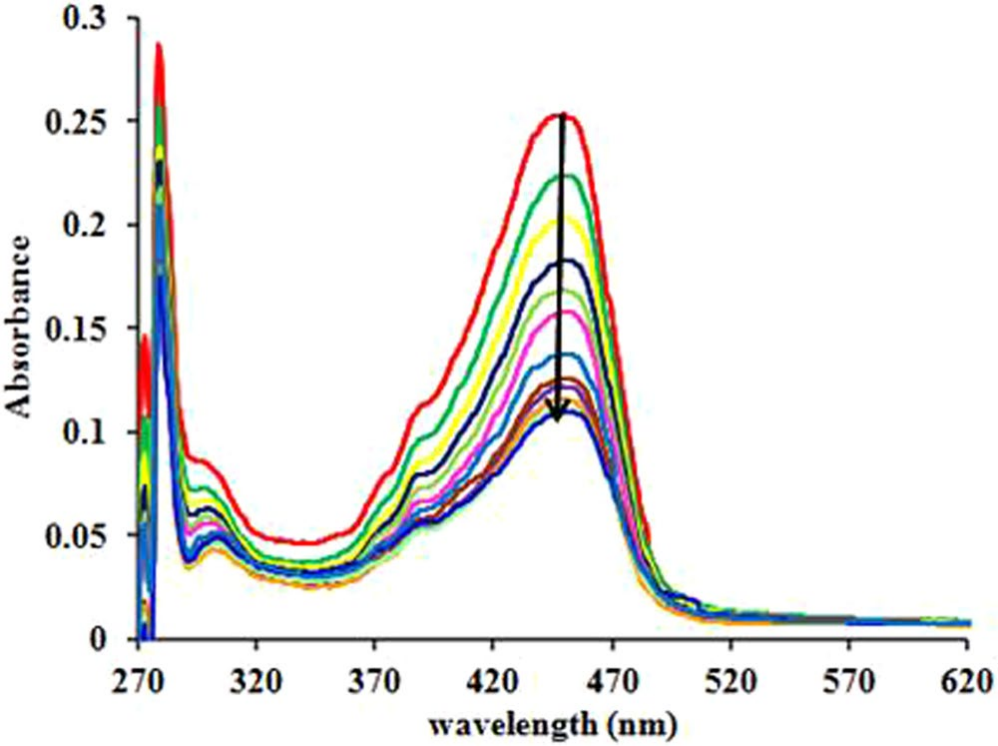
Change in the UV-Vis spectra of compound (*S*)-**3a** (10 µM, red trace) on incremental addition of COX-2.

The UV-Vis spectrum of compound (*S*)-**3a** at 10 µM concentration in Tris-HCl buffer (pH 7.25) exhibited absorption bands at 279 and 450 nm. The incremental addition of COX-2 to the solution of compound (*S*)-**3a** resulted in the decrease in absorbance at 279 and 450 nm indicating the interactions of compound with COX-2 (Fig. 4). The changes in the UV-Vis spectra of (*S*)-**3a** on addition of COX-2 are due to π-π and hydrophobic interactions between compound (*S*)-**3a** and COX-2. The binding constant between compound 6 and COX-2 was determined using Benesi-Hildebrand Equation^28^ (equation 1)

1 $$1/({{\rm{A}}}_{{\rm{f}}}\,-\,{{\rm{A}}}_{{\rm{obs}}})=1/({{\rm{A}}}_{{\rm{f}}}\,-\,{{\rm{A}}}_{{\rm{fc}}})+1/{{\rm{K}}}_{{\rm{a}}}({{\rm{A}}}_{{\rm{f}}}\,-\,{{\rm{A}}}_{{\rm{fc}}})[{\rm{L}}]$$

Where A_f_ is absorbance of free host, A_obs_ is absorbance observed, A_fc_ is absorbance at saturation, K_a_ is binding constant and [L] is Ligand concentration. Compound (*S*)-**3a** showed significant interactions with COX-2 with binding constant, K_a_ 5.36 × 10^5^ M^−1^.

### NMR Chemical Shift and Relaxation Experiments

**Figure 5:**
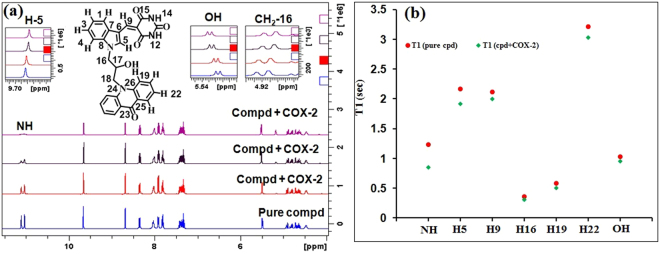
(**a**) ^1^H NMR spectra of compound (*S*)-**3a** in the absence (blue trace) and presence of COX-2 (red, green and purple trace). Inset: expansion of a part of spectrum showing change in chemical shifts on incremental addition of COX-2. (**b**) ^1^H T1 Relaxation times of compound (*S*)*-***3a** (15 mM) in the presence and absence of enzyme COX-2.

The interactions between the enzyme and compound (*S*)-**3a** were further supported^29^ by recording changes in the NMR chemical shifts and T1 of the compound in the presence of COX-2. ^1^H NMR spectra of (*S*)-**3a** (15 mM) in deuterated dimethyl sulphoxide (DMSO-*d*_6_) at 25 °C were recorded in the absence (blue trace, Fig. 5a) and in the presence of COX-2 (red, green and purple trace; Fig. 5a, Fig. S166–S169). It was observed that on incremental addition of COX-2 to the solution of compound (*S*)-**3a**, there is sharp decrease in the intensity of NH signals at δ 11.06 and 11.12 ppm whereas the signals of H5 (9.66 ppm) and C-16H_2_ (δ 4.90–4.93 ppm) were shifted upfield. These changes in the intensity and chemical shifts of the compound were mainly attributed to its NH– and hydrophobic– interactions with the enzyme. Downfield chemical shift of OH signal at δ 5.5 ppm pointed towards the possibility of H-bond interactions between the compound and amino acid residues (Fig. 5a) and the same interaction was visible in the molecular docking image (Fig. 1b,c). The decrease in the intensity of NH signal at 11 ppm has probably occurred due to the abstraction of NH proton by the guanidine moiety of R120 as this group is engaged in salt bridge formation (Fig. 1b,c). The binding of compound with the enzyme was also confirmed by T_1_ experiments where spin-lattice relaxation time (T_1_) of the Hs’ of compound (*S*)-**3a** was measured (Fig. 5b). Characteristically, supporting the chemical shifts data and molecular docking studies and confirming the hydrophobic and H-bonding interactions of compound (*S*)-**3a** with COX-2, the relaxation time of NH, H5, H9, H16, H19, H22 and OH protons was considerably decreased in the presence of COX-2.

Therefore, before proceeding for the complicated and expensive biological experiments, the physico-chemical data obtained from molecular docking studies, ITC, UV-vis and NMR experiments helped to identify molecule (*S*)-**3a** for its appreciable interaction with COX-2. Moreover, the consonance of molecular docking results with NMR experimental results pin-pointed the part of (*S*)-**3a** involved in hydrophobic and H-bond interactions with the enzyme.

## Biological Experiments

### Screening of the compounds for COX-1, COX-2 and 5-LOX inhibitory activities

Although the results of physico-chemical experiments indicated that compound (*S*)-**3a** exhibits desirable interactions with COX-2 but still to make comparison, we included all the compounds 3–4 for enzyme immunoassays. The enzyme immunoassays were performed in triplicate at five different concentrations of the compounds and the results given in Table 2 are the average of the three experiments. Principally, the catalytic activity of COX-1/2 is slowed down in the presence of its inhibitor and quantitatively, it is inversely proportional to the amount of prostaglandin produced in the presence of inhibitor. Hence, the efficacy of the compounds was measured by calculating the amount of prostaglandins that was synthesized by the enzymes in the presence of the compounds.

**Table 2:** IC_50_ (µM) of compounds 3–4 for COX-1 and COX-2. SI = IC_50_ (COX-1)/ IC_50_ (COX-2).

Compound	IC_50_ (µM)		Selectivity index	IC_50_ (µM)
	COX-2	COX-1		5-LOX
1	0.7 ± 0.01	118 ± 5.5	168.5	—
*(S)*−2	0.001 ± 0.0008	0.34 ± 0.025	340	0.0015 ± 0.0007
*(R)*−2	7 ± 0.45	54 ± 2.5	7.7	—
*(S)*-**3a**	0.0006 ± 0.0001	1.0 ± 0.12	1666	0.2 ± 0.015
*(R)*-**3a**	0.4 ± 0.16	>10	>25	1.0 ± 0.1
*(S)*-3b	0.3 ± 0.18	0.7 ± 0.05	2.33	0.09 ± 0.012
*(R)*-3b	0.1 ± 0.07	1.0 ± 0.2	10	2.0 ± 0.18
*(S)*-3c	0.8 ± 0.075	60 ± 3	75	0.5 ± 0.028
*(R)*-3c	80 ± 7	8 ± 0.38	0.1	0.6 ± 0.042
*(S)*-3d	6 ± 0.5	60 ± 4.5	10	0.6 ± 0.039
*(R)*-3d	—	>10	—	0.1 ± 0.06
*(S)*-3e	—	—	—	0.7 ± 0.038
*(R)*-3e	nd	nd	nd	0.3 ± 0.13
4a	0.1 ± 0.06	30 ± 1.8	300	0.2 ± 0.01
4b	8.0 ± 0.42	>10	>1.25	0.07 ± 0.022
4c	0.3 ± 0.015	4.0 ± 0.32	13.33	0.1 ± 0.08
4d	3.0 ± 0.22	4.0 ± 0.18	1.33	0.2 ± 0.09
4e	9.0 ± 0.55	>10	>1.11	0.4 ± 0.015
4f	40 ± 3.8	—	—	0.1 ± 0.07
Indomethacin	0.96	0.08	0.08	—
Diclofenac	0.02	0.07	3.5	—
Celecoxib	0.04	15	375	—
Zileuton	—	—	—	0.3

Compound (*S*)-**3a** displayed significant inhibition of COX-2 with IC_50_ (50% inhibitory concentration) 0.6 nM and also exhibited good selectivity index over COX-1 as its IC_50_ for COX-1 was 1.0 µM. Compound (*S*)-3b and (*S*)-3c displayed COX-2 inhibition with IC_50_ values 0.3 and 0.8 µM but their selectivity index for COX-2 over COX-1 was low. It was noticed that the *R*- enantiomers of compound **3a**–3d exhibit less enzyme inhibitory activity in comparison to the corresponding *S*- enantiomers except in case of compound 3b (Table 2). We did not get significant results with compound 3e. Excitingly, the COX-2 inhibitory activity of compound (*S*)-**3a** was found improved over that of compound (*S*)−2.

Compounds 4a-f having alkyl linker between acridone and indole moieties also showed good to moderate inhibition of COX-2 but the selectivity index for COX-2 was poor (Table 2). In parallel to the results of physico-chemical experiments, significant difference in the enzyme inhibitory activity of compound (*S*)-**3a** and 4a indicates that the linker between acridone and indole play specific role in the enzyme – inhibitor interaction. Moreover, the effect of the stereocentre on the efficacy of compound (*S*)-**3a** and (*R*)-**3a** points towards the non-promiscuous nature of the compounds and rules out the possibility of their frequent hitters. Hence, the problem of pan assay interference seems insignificant for these compounds. Compound (*S*)-**3a**, (*S*)-3b and (*S*)-3c also exhibited moderate IC_50_ values 0.2, 0.09 and 0.5 µM for lipoxygenase (5-LOX). As did in one of our previous studies^22^, the enzyme immunoassay was also performed in the presence of Triton where we did not observe changes in the COX-2 inhibitory activity of the compounds. This observation ruled out the possibility that the compounds do not work through aggregation induced inhibition of the enzymes. From the enzyme immunoassays, we identified compound (*S*)-**3a** as an appreciable inhibitor of COX-2 and it was further subjected to pharmacokinetic (PK) studies and tested for anti-inflammatory and analgesic activity over the animal models.

### Human Whole Blood Assay for COX-1 and COX-2

**Figure 6:**
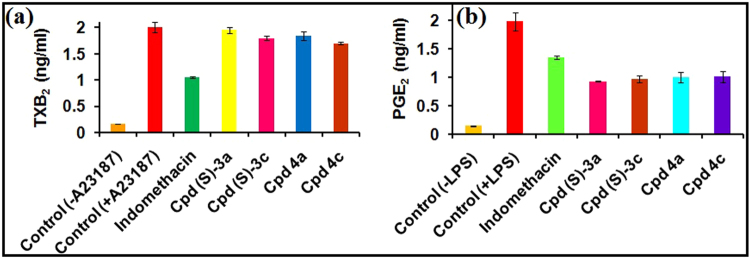
Inhibition of (**a**) TXB_2_ in calcium ionophore (A23187) stimulated whole blood and (**b**) PGE_2_ in LPS stimulated whole blood by indomethacin, (*S*)-**3a**, (*S*)-3c, 4a, and 4c (final conc 1 µM).

The use of human blood for the whole blood assay was duly approved by the institutional ethical committee of the University for human subjects. Production of prostaglandin PGE_2_ during lipopolysaccharide (LPS) stimulated whole blood was used to quantify COX-2 inhibitory activity whereas calcium ionophore (A23187) stimulated thromboxane (TxB_2_) production was used to measure COX-1 inhibitory activity of the compounds. A23187-Stimulation of human whole blood resulted in increase in TxB_2_ production when compared with control treated blood (Table 3). Administration of 1 µM of compound (*S*)-**3a** had no effect on ionophore stimulated TxB_2_ production, indicating compound (*S*)-**3a** exhibited almost no inhibition of COX-1. Similarly, LPS-stimulation of human whole blood resulted in increase in PGE_2_ production compared with control treated blood (Table 3). Administration of 1 µM of compound (*S*)-**3a** inhibited LPS stimulated PGE_2_ production thus confirming the results of COX-2 selectivity observed in the enzyme immunoassays. Similar results were observed in case of other tested compounds (Table 3, Fig. 6). In contrast to the results of enzyme based assay, the similarity in the COX-2 selectivity and inhibition of compound **3a** and 3c was probably due to the use of higher molar concentration (1 μM) of these compounds.

**Table 3:** Calcium ionophore stimulated inhibition of TXB_2_ and LPS stimulated inhibition of PGE_2_ in whole blood cells by compounds (S)-**3a**, (S)-3c, 4a, and 4c (final conc 1 µM).

	Production of TXB_2_ (ng/mL)		Production of PGE_2_ (ng/mL)	
	−Ca^2+^ Ionophore	+Ca^2+^ Ionophore	−LPS	+LPS
Control	0.16 ± 0.09	2.00 ± 0.14	0.145 ± 0.07	1.98 ± 0.12
Indomethacin		1.05 ± 0.25		1.35 ± 0.4
(*S*)-**3a**		1.95 ± 0.01		0.93 ± 0.1
(*S*)-3c		1.80 ± 0.5		0.97 ± 0.09
4a		1.84 ± 0.11		1.00 ± 0.13
4c		1.70 ± 0.3		1.01 ± 0.14

### Pharmacokinetic profile of compound (S)-**3a**

**Figure 7:**
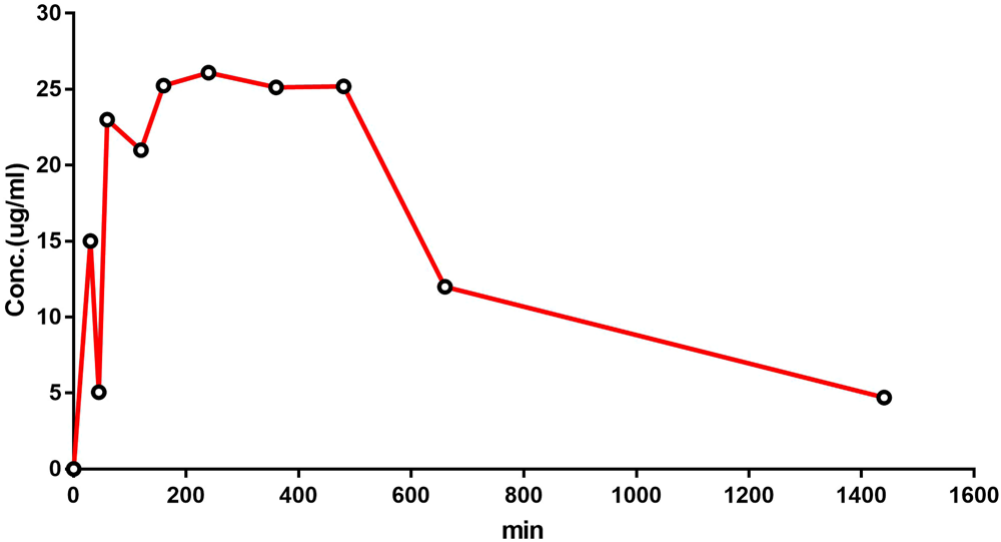
Pharmacokinetic profile for compound (*S*)-**3a** in rats.

10 mg kg^−1^ dose of compound (*S*)-**3a** was administered intraperitoneally (ip). The blood samples were withdrawn from jugular vein at an interval of 30, 45, 60 min and 2, 3, 4, 6, 8, 11, 24 h. The plasma fraction was obtained by centrifugation at 4 °C, 8000 rpm for 6 min and stored at −20 °C. The samples for LC-MS were prepared by using protein precipitation method. The compound demonstrated half-life ~7.5 h and C_max_ 26.1 μg/mL (Table 4, Fig. 7). Since after 24 h, appreciable concentration of the compound was observed in the serum, it may be inferred that the compound may act for a longer time.

**Table 4:** Rat pharmacokinetic profile of compound (*S*)-**3a**.

Parameter	Unit	Value
Dose level	mg/kg	10
t_1/2_	min	453.9041712
T_max_	min	240
C_max_	μg/ml	26.1
AUC 0-t	μg/ml*min	20837.5
AUC 0-inf_obs	μg/ml*min	23915.4729
MRT 0-inf_obs	min	704.4877033

### Analgesic and anti-inflammatory activity of compound (S)-**3a**

*In-vivo* biological experiments were performed on male wistar rat (250–300 g). The animals were kept in the animal house under 12 h light/12 h dark cycle maintaining the temperature 22 ± 2 °C. The animals were given free supply of food and water. The use of animals for the biological studies was duly permitted by the institutional animal ethical committee (IAEC) of the University. Acetic acid induced algesia model and carragennan induced inflammatory model were used for these studies. For studying the mode of action of the compound; the animals were administered Substance P, L-arginine, L-NAME and A23187 prior to the compound (*S*)-**3a**. All the doses were administered intraperitoneally. A schematic representation of the protocols of these studies is shown in the supporting information (Fig. S170–S172) and the details are given in the experimental section.

**Figure 8:**
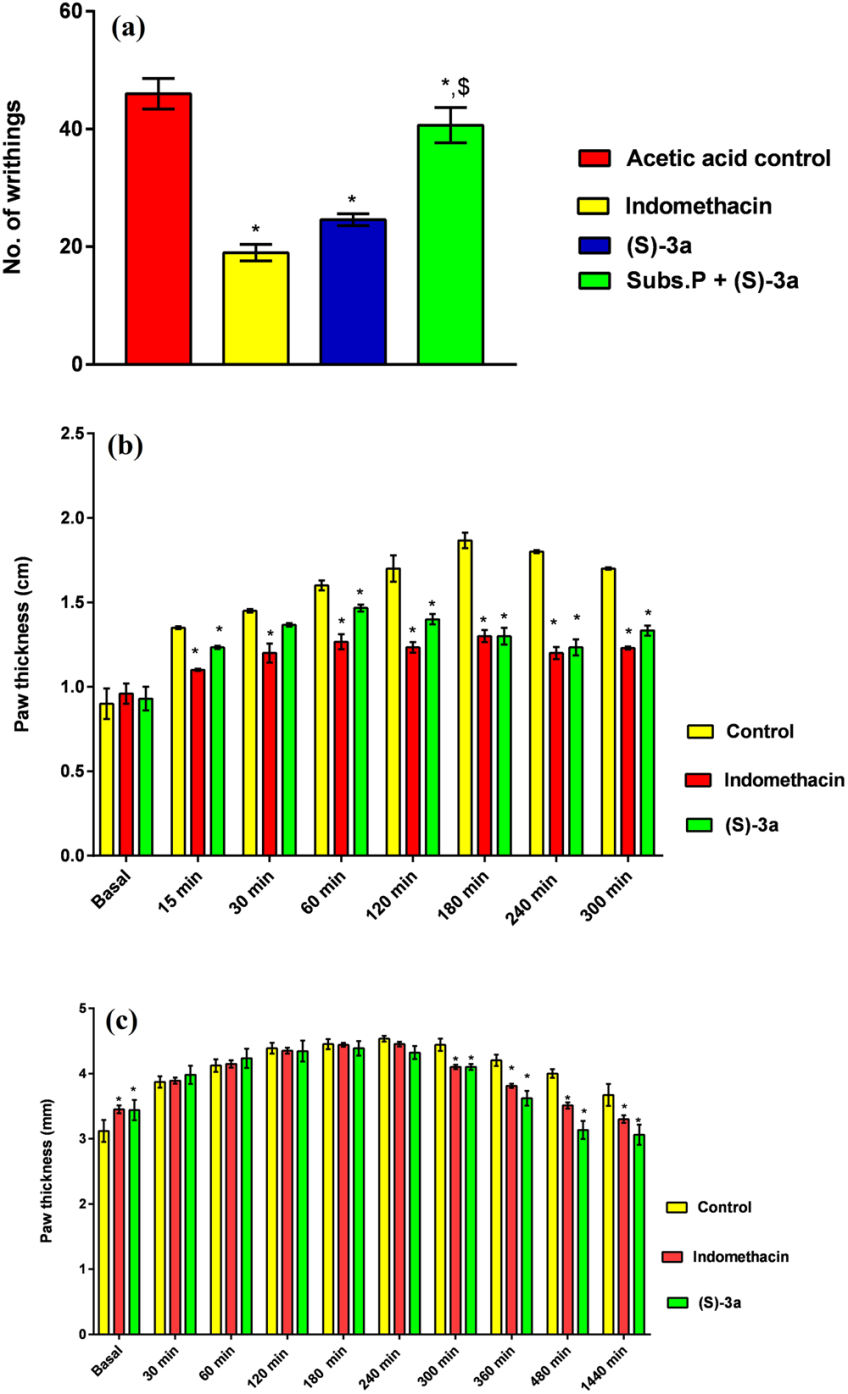
Effect of compound (*S*)-**3a** (**a**) on acetic acid induced writhings in rat and (**b**) carrageenan-induced inflammation in rat. All values are expressed as mean ± SEM. *p < 0.05 vs control. (**c**) Effect of compound (*S*)-**3a** after carageenan induced paw edema. All values are expressed as mean ± SEM. *p < 0.05 vs control.

In comparison to the control group of animals; the administration of indomethacin (used as the standard drug) and compound (*S*)-**3a** to the rat significantly decreased the number of acetic acid induced writhing. Compound (*S*)-**3a** decreased the number of writhing by 50% (Fig. 8a).

Compound (*S*)-**3a** was further screened for the treatment of carageenan induced inflammation in rat. The animals were administered vehicle, indomethacin, compound (*S*)-**3a** intraperitoneally, and after 30 min of the dose, inflammation was induced with carageenan. The paw thickness of the animals was measured with vernier callipre at 1 h intervals up to the 5^th^ hour and compared with the control. Significant decrease in the carageenan induced paw inflammation was observed in case of animals pretreated with compound (*S*)-**3a**. At the end of 4^th^ and 5^th^ hour, the effect of the compound was comparable to that of indomethacin (Fig. 8b).

### Effect of compound (S)-**3a** on pre-existing inflammation

To evaluate the curative effect of the pharmacological interventions in carrageenan induced inflammation, compound (S)-**3a** and indomethacin were given at doses 10 mg kg^−1^ intraperitonially 60 min after the carrageenan injection. Paw thickness (mm) was used as an index of inflammation and measured at 30 min, 1 h, 2 h, 3 h, 4 h, 5 h, 6 h, 8 h, and 24 h after the carrageenan injection. Treatment with indomethacin and compound (*S*)-**3a** was found to decrease paw edema after 5 h of treatment. The decrease in paw edema was sustained even after 24 h of treatment (Fig. 8c).

### Mechanistic Studies

**Figure 9:**
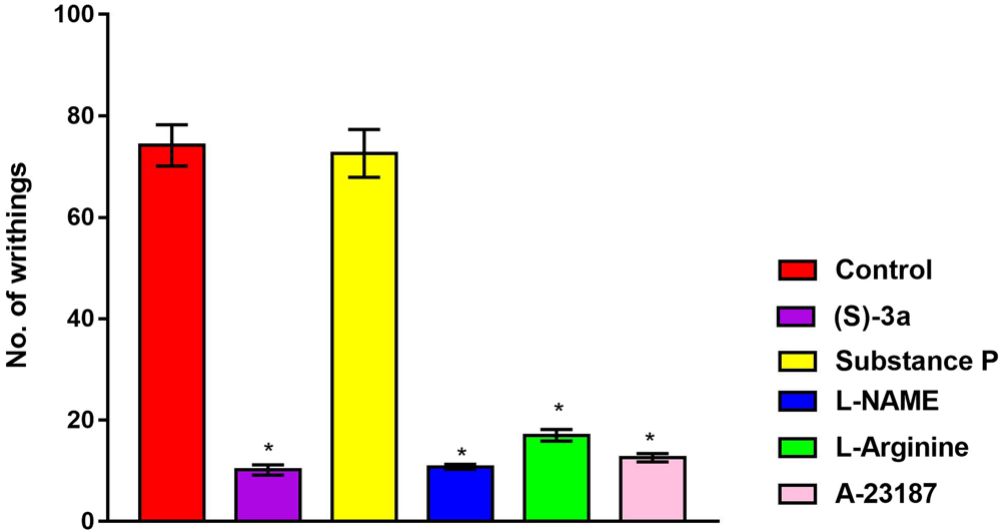
Effect of substance P, L-arginine, L-NAME and A-23187 on the analgesic effect of compound (*S*)-**3a**. All values are expressed as mean ± SEM. *p < 0.05 vs. control.

For exploring the possible involvement of COX, LOX, nitric oxide pathways and calcium channel blocker in the mode of action of compound (*S*)-**3a**, the animals were pretreated with substance P, L-arginine, L-NAME and A23187, respectively (Fig. 9). Substance P is known for activating COX and LOX^30^. Pretreatment of the animals with substance P attenuated the analgesic effect of the compound whereas L-arginine, L-NAME and A23187 did not affect the action of compound (*S*)-**3a**. Therefore, along with the results showing selectivity of (*S*)-**3a** for COX-2 over COX-1, the experiments for mechanistic studies indicated that the analgesic effect of compound (*S*)-**3a** might be operating through the inhibition of COX-2 and LOX pathway.

Therefore, supporting the findings of physico-chemical experiments, the battery of biological experiments confirmed that compound (*S*)-**3a** targets COX-2 and possesses sufficient potential to reverse analgesia and inflammation in the animal model and hence this molecule may act as lead molecule to anti-inflammatory drug.

### Acute Toxicity Studies

**Figure 10:**
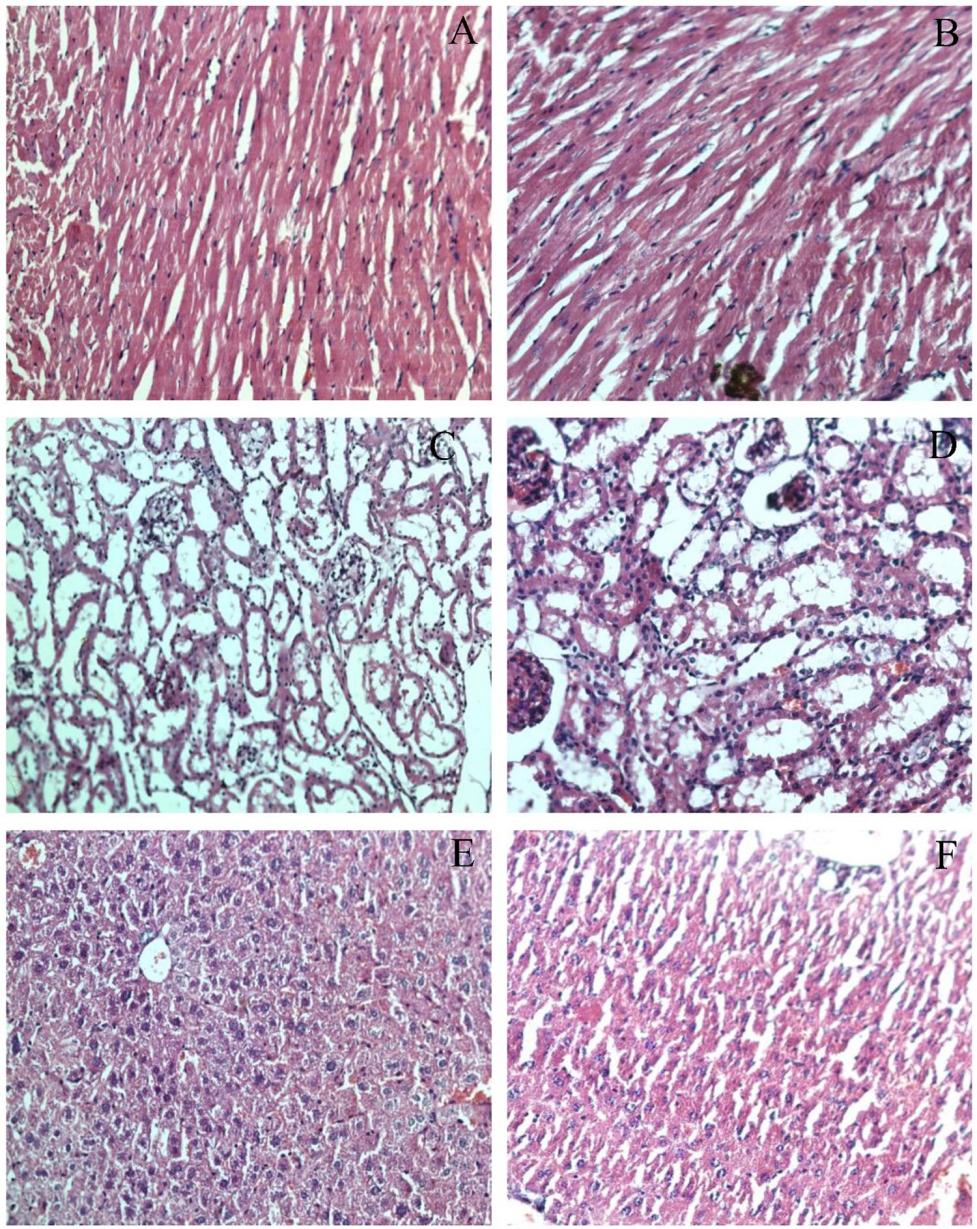
Histology of myocardium of control (**A**, 20x) and compound (*S*)-**3a** treated (**B**, 20x); kidney of control (**C**, 20x) and compound (*S*)-**3a** treated (**D**, 20x); liver of control (**E**, 20x) and compound (*S*)-**3a** treated (**F**, 20x) mice.

**Figure 11:**
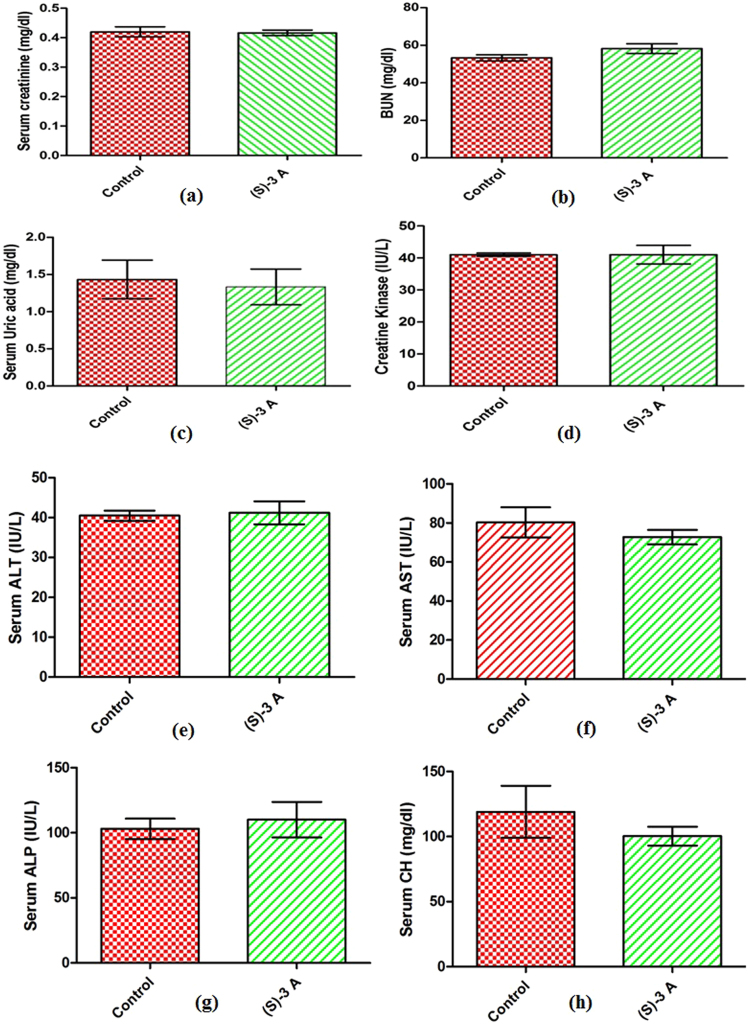
(**a**) Effect of compound (*S*)-**3a** on renal function tests in acute toxicity studies in mice. All values expressed as mean ± SEM. (**b**) Effect of compound (*S*)-**3a** on liver function tests in acute toxicity studies in mice. All values expressed as mean ± SEM.

Using the previously described procedure^22^, the acute toxicity of compound (*S*)-**3a** was checked. The animals were divided into four groups of 3 animals each. The first group of animals was taken as control and it was administered vehicle. Compound (*S*)-**3a** at doses of 50 mg kg^−1^, 300 mg kg^−1^ and 2000 mg kg^−1^ was given to the second, third and fourth group of animals, respectively. As per the OECD guidelines, the compound was administered orally using a stomach tube. No gross behavioural abnormality or mortality was observed in any of the groups in the 14 days of the study. The histological sections revealed no remarkable alterations in the compound treated group as compared to the control group (Fig. 10, Fig. S173). Moreover, the treatment with compound (*S*)-**3a** at 2000 mg kg^−1^ did not produce any significant alterations in the levels of serum creatinine, blood urea nitrogen (BUN), uric acid, creatinine kinase, alanine aminotransferase (ALT), aspartate aminotransferase (AST), alkaline phosphatase (ALP) or cholesterol as compared to untreated animals (Fig. 11). We did not observe changes in the body weight of the treated animals as compared to the normal animals and also the ratio of kidney weight to body weight was not significantly different in both the groups (Fig. S174).

Therefore, starting with the molecular modelling studies, the modification of one of our previously reported molecules provided a highly potent inhibitor of COX-2. Appreciable interactions of compound (*S*)-**3a** with COX-2 were recorded with ITC, UV-vis and NMR experiments. Compound (*S*)-**3a** exhibited IC_50_ 0.6 nM for COX-2 and its selectivity index for COX-2 over COX-1 was 1666. It was found that this compound is capable of reducing analgesia and inflammation in rat by 50% and 25% respectively when administered at 10 mg kg^−1^ dose. Compound (*S*)-**3a** also exhibited desired toxicity and PK profile with maximum tolerable dose (MTD) 2000 mg kg^−1^ and t_1/2_ 7.5 h. The specificity of the enzyme active site was apparent from the incapability of the *R*- enantiomer of **3a** to inhibit the enzymatic activity of COX-2. Overall, the idea of introducing a large hydrophobic moiety in the form of acridone in compound (*S*)−2a worked satisfactorily and a combination of physico-chemical and biological experiments helped to identify a new lead molecule (*S*)-**3a** to the anti-inflammatory drug.

## Method

### General Note

Melting points of the compounds were determined in capillaries. NMR spectra of the compounds were recorded on JEOL 400 MHz, 100 MHz and Bruker 500 MHz and 125 MHz NMR spectrometer, respectively. Deuterated chloroform and deuterated dimethyl sulphoxide were used as solvents. Chemical shifts are mentioned in parts per million (ppm) w.r.t. tetramethylsilane (TMS) (internal reference). Coupling constants (*J*) are given in Hertz (Hz). Bruker micrOTOF QII Mass Spectrometer was used for recording high resolution mass spectra (HRMS). The reactions under microwave conditions were performed in a microwave synthesizer (BIOTAGE INITIATOR EXP - RU) at 90 W and 150 °C. Glass plates coated with silica gel GF-254 were used for thin layer chromatography (TLC) for the monitoring of the reactions. For column chromatography, 60–120 mesh silica was used. UV-vis spectra were recorded on BIOTEK Synergy H1 Hybrid Reader instrument. Optical rotation was recorded on ATAGO POLAX-2L polarimeter at 25 °C in DMSO with wavelength 589 nm. High performance liquid chromatography (HPLC) was performed on Shimadzu LC-20 AD instrument. 98–99% purity of the compounds was ascertained with q^1^HNMR technique^31^ (Fig. S133, supporting information and accompanying calculation). All *in-vivo* experiments were performed as per relevant guidelines and regulations and were approved by the institutional ethical committees: Institutional Animal Ethical Committee (IAEC) of Guru Nanak Dev University, Amritsar and Institutional Ethics Committee of Guru Nanak Dev University Amritsar for Human Subjects.

### General procedure

An equivalent mixture of appropriate aldehyde (1 mmol) and barbituric acid/1, 3-dimethyl barbituric acid/1-(3-chlorophenyl)-3-methyl-2-pyrazolin-5-one (1 mmol) in dimethyl formamide (2 mL) was subjected to microwave irradiations at 150 °C for 1 h. The reaction was monitored with TLC (eluent for TLC: Ethyl acetate - Hexane (80:20). After the completion, the reaction was quenched by adding water to the reaction mixture. The crude product was separated out as solid, which was further purified with ethanol and hot water washings and finally recrystallized from chloroform:methanol (9:1).

### Screening of inhibitory activities of the compounds against COX-1, COX-2 and 5-LOX

The enzyme inhibition screening assay kits for COX-1/2 and 5-LOX with item no. 560131 and 760700, respectively were purchased from Cayman Chemical Co. The solutions of compounds were prepared in DMSO at 10^−5^–10^−9^ M concentrations. For compound (*S*)-**3a**, enzyme immunoassay was repeated with 10^−6^ M – 10^−10^ M concentrations of the compound. For COX reactions; background tubes were prepared by taking 10 µL heme and 10 µL of inactive enzymes (enzymes were inactivated by placing enzymes containing microfuge tubes in boiling water) in 160 µL reaction buffer. The 100% initial activity tubes for the two enzymes contained 10 µL heme and 10 µL of the enzyme in 160 µL reaction buffer. Then 10 µL inhibitor (compound of above mentioned concentrations) was added to the inhibitor tubes for the two enzymes and 10 µL of vehicle (DMSO) was added to 100% initial activity and background tubes. All the tubes were incubated for 10 min at 37 °C and the reaction was started by adding 10 µL arachidonic acid to all the reaction tubes. After incubation of the reaction vials for 2 min at 37 °C, the enzyme catalysis was stopped by adding 30 µL saturated stannous chloride solution to each test tube and solutions were kept at 0–4 °C. For EIA (enzyme immunoassay) procedure: the EIA buffers and assay specific reagents were prepared as written in the manual. In the COX dilutions, two test tubes labelled as BC1 and BC2 were taken to prepare background samples. Each tube contained 990 µL of EIA buffer and 10 µL of background COX-1 for BC1 and 10 µL of background COX-2 for BC2. The samples for 100% initial activity were diluted in three vials labelled IA1, IA2 and IA3 for each enzyme. IA1 contained 990 µL EIA buffer and 10 µL enzyme, IA2 has 950 µL buffer and 50 µL solution of IA1 (1:2000 dilution of original sample) and IA3 carried 500 µL of buffer and 500 µL of IA2 solution (1:4000 dilution of original sample). Each solution was thoroughly mixed. Solutions of IA2 and IA3 were used for the assaying. Similar to the 100% initial activity dilutions, the inhibitor samples were diluted in vials labelled C1–C3. Vial C1 has 990 µL EIA buffer and 10 µL sample from inhibitor tubes. C2 contained 950 µL buffer and 50 µL solution of C1 (1:2000 dilution of original sample) and vial C3 was charged with 500 µL buffer and 500 µL solution from C2 (1: 4000 dilution of original sample). Only C2 and C3 were run in the assay. After all the reaction dilutions, these were then subjected to 96 wells plate. The wells were divided as: 2 blank wells, 2 NSB wells (non-specific binding), 3 B_0_ wells (maximum binding), one TA well (total activity), 16 wells for standards (S1–S8 in repeat), 2 BC1 and 2 BC2 wells. Compounds (inhibitors) were put in all the other wells. The sequence for the addition of reagents onto the plate was as per the protocol of the assay kit. Covered with plastic film, the plate was left for incubation for 18 h at room temperature. For developing the plate; it was emptied and washed with wash buffer 5 times followed by the addition of 200 µL Ellman’s reagent to each well. 5 µL tracer was added to the TA well. Then the plate was covered and left for 60 mins for development in dark. After the respective time period the cover was removed and the plate was read at 420 nm. %B/B_o_ (i.e. % sample or maximum bound) value for standards S1-S8 and each sample was determined from the absorbance values obtained according to the calculation given in the protocol. The amount of prostaglandins formed during the enzymatic reaction was calculated from the values of %B/B_o_ for all the compounds at all concentrations with the help of the standard curve (plotted between %B/B_o_ of standards and raw data given in the protocol). Then the prostaglandin formed at each concentration of all the compounds and percentage inhibition values were calculated. The graph between percent inhibition of the enzyme and corresponding concentration of the compound provided IC_50_ values for the compounds.

### Lipoxygenase inhibitory activity assay

Five concentrations of the compounds (10^−8^ M–10^−4^ M) were prepared in DMSO and tested in duplicate. 10 µL of the compound of each concentration was mixed with 90 µL 5-LOX (Soybean lipoxygenase in assay buffer) in the specified wells of the 96-well plate. Two wells containing assay buffer and AA acted as blanks whereas four wells, each carrying enzyme and AA, were positive controls. 10 µL AA was added to each test well and the plate was shaken for 5 min. Chromogen (100 µL) was added to each well and the plate was again shaken for 5 min. The absorbance of each well at 490 nm was recorded on microplate scanning spectrophotometer. As per the protocol given with the assay kit, the 5-LOX inhibitory activity for each compound at each concentration was determined using the mean of the two values (duplicate experiments) with deviation <5%.

### Human Whole Blood Assay

#### Sample Preparation

Approval for the use of human blood was taken from the institutional ethical committee of Guru Nanak Dev University Amritsar and informed consent was obtained from the subject^32,33^. All experiments were performed in accordance with the relevant guidelines and regulations. By venipuncture, the blood sample was collected in heparinized tubes. For performing COX-1 assay; 100 µL of the compound/ indomethacin/ vehicle (DMSO) (final conc 1 µM) was added to 300 µL of blood aliquots and kept for 60 min. Subsequently, 100 µL calcium ionophore, A23187 (final conc 50 µM) was added and the samples were kept for 30 min. Plasma was removed by centrifugation (1500 × g, 4 °C, 5 min) and was immediately frozen. Each sample in duplicate was assayed for TxB_2_ (the breakdown product of TxA_2_) using TXB_2_ Express ELISA kit (Cayman Chemicals, Ann Arbor, MI) by the reported protocol^34^.

COX-2 assay was performed by inactivating COX-1 with 50 µL aspirin (10 µg/mL) per 400 µL of blood sample. The incubation of the samples for 6 h was followed by the addition of 100 µL of test compound/indomethacin/ vehicle (DMSO) (final conc 1 µM) and the samples were kept for 15 min. 50 µL LPS solution (final concentration = 10 µg mL^−1^) was added and the samples were incubated for another 18 h. Plasma was removed by centrifugation (1000 × g, 4 °C, 15 min) and stored at −20 °C. Following the reported protocol^34^, COX inhibitor screening assay kit was used for quantifying PGE_2_.

### *In-vivo* experiments

The use of animals for the *in-vivo* experiments was duly approved by the institutional animal ethical committee (IAEC) of Guru Nanak Dev University, Amritsar.

### Pharmacokinetic studies

#### *In-vivo* pharmacokinetic studies

The *in vivo* pharmacokinetic properties were evaluated using male wistar rat (250–300 g). The compound at 10 mg kg^−1^ dose was suspended in 0.1% CMC and administered intraperitonealy to the rats. The animals were anesthetized with ketamine (50 mg kg^−1^ i.p). The blood samples were withdrawn from jugular vein at an interval of 30, 45, 60 min and 2, 3, 4, 6, 8, 11, 24 h of drug administration and collected in heparinised tubes. Samples were withdrawn in triplicate (3 samples/time interval). 100 μL of blood sample was withdrawn at each interval from one animal. The plasma fraction was obtained by centrifugation at 4 °C, 8000 rpm for 6 min. The samples were stored at −20 °C. The samples for LC-MS were prepared by using protein precipitation method. 100 μL plasma sample was taken in 1.5 mL tube and vortex for 3 min. 300 μL acetonitrile with internal standard was added to the above tube and vortex for 5 min. The contents of the tube were centrifuged at 4 °C, 16000 rpm for 40 min. The compound with initial concentration 3 mg mL^−1^ followed by serial dilution was used for obtaining standard curve. LC-MS was performed with Dionex ultimate 3000 HPLC system attached to Bruker MicroTof QII mass spectrometer. 50 mm, 5 μm PRP C18 column was used for HPLC and the gradient mobile phase was consisting of water and acetonitrile (each containing 0.1% formic acid). The initial composition was 20% acetonitrile and linearly increased to 100% in 30 min. The column eluent was introduced to the ESI source of mass spectrometer operating in +ve mode. The different pharmacokinetic parameters such as t_½_ lives (min), AUC (area under curve), C_max_ (µg/ml), t_max_ (min), mean residence time (min) were determined following non compartmental analysis in PK solver software^35^.

### *In-vivo* studies on animal models

#### Evaluation of Analgesic and anti-inflammatory Activity

Analgesic activity was studied using acetic acid induced writhing. Animals in 13 groups, each consisting of 5 animals, were used. Intraperitoneal administration of 0.6% acetic acid induced abdominal writhings that were characterized by arching of back, extension of hind limbs and contraction of abdominal musculature^36^. For checking the involvement of cyclooxygenase and lipooxygenase pathway in the mode of action of the compound, Substance P was used whereas L-arginine and L-NAME were used to investigate the involvement of nitric acid pathway. The role of Ca^++^ was studied by A23187 pretreatment as previously described^34^. For anti-inflammatory activity, carageenan induced paw edema model with three groups of animals, 5 in each, were used^22^. All the drugs were administered intraperitoneally.

### Studying the therapeutic effect

To evaluate the curative effect of the pharmacological interventions in carrageenan induced inflammation, 10 mg kg^−1^ of compound (*S*)-**3a** and indomethacin were given i.p 60 min after carrageenan injection. Paw thickness (mm) was used as index of inflammation and measured at 30 min, 1 h, 2 h, 3 h, 4 h, 5 h, 6 h, 8 h, and 24 h after the carrageenan injection.

#### Studying the Acute Toxicity of the compound

The toxicity studies, as previously described^22^, were performed on the most active compound. Four groups of animals, 3 in each group, were taken. The first group of animals was administered vehicle and acted as control. 50 mg kg^−1^, 300 mg kg^−1^ and 2000 mg kg^−1^ of compound (*S*)-**3a** was given to the second, third and fourth group of animals. The compound was suspended in 0.1% CMC and given orally. Prior to the dosing, the animals were fasted for 4 h and after dosing, the animals were kept under continuous observation for the first four hour. For the next 14 days, the animals were observed daily and then sacrificed. The morphological changes were observed in the viscera. The histological studies were carried out using H & E staining.

### Histopathological and Biochemical tests

On 14^th^ day the animals were anaesthetized with ketamine (50 mg/kg, i.p) and blood was withdrawn by cardiac puncture, thereafter the animals were sacrificed. The liver and kidney tissues were isolated, kept in 10% formalin and subjected to histopathological examination. Blood was centrifuged at 5,000 rpm at 4 °C for 10 min to isolate serum. Serum samples were subjected to biochemical analysis for the liver and renal function tests. Serum creatinine, blood urea nitrogen (BUN), uric acid, creatine kinase, alanine aminotransferase (ALT), aspartate aminotransferase (AST), alkaline phosphatase (ALP), and cholesterol levels were estimated using commercially available kits.

#### Studying the mode of action of compound (S)-**3a**

As described in the previous studies^34^, animals were pretreated with substance P for checking the involvement of cyclooxygenase and lipooxygenase pathway and with L-arginine and L-NAME for checking the participation of nitric oxide pathway during the inhibition of algesia. For investigating the involvement of calcium channel, the animals were pretreated with A23187.

### UV-vis Spectral Studies

Solution of compound (10 µM) was prepared in HPLC grade DMSO and Tris-HCl buffer (1:9) (pH 7.25). 3 µL of enzyme (3 μM) was diluted with 100 µL of Tris-HCl buffer. UV-vis spectra of the compound were recorded after every addition of 5 μL of the enzyme until 60 μL of enzyme was added. The decrease in absorbance intensity of the compound on addition of enzyme solution was checked with the control experiment where no decrease was observed on incremental addition of buffer up to 60 μL.

### Isothermal Titration calorimetric experiments

100 µM solution of the compound in HPLC grade DMSO and Tris-HCl buffer (1:9) (pH 7.4) was prepared. 5 µL of enzyme was diluted with 500 µL Tris-HCl buffer. 19 consecutive injections of 2 µL each at 120 s interval were made to the enzyme solution taken in the sample cell. The heat change in the cell was determined. The control titrations for determining the background heat (that was subtracted from the main experiment) were performed by taking buffer solution in the cell and making successive additions of the titrant. The titration heat profiles and the binding parameters were read with Microcal software origin 7.0.

### NMR studies for Protein-Ligand interactions

NMR experiments were performed on Bruker Avance 500 NMR spectrometer at 298.2 K.

#### ^1^H NMR T_1_ relaxation time measurements

The longitudinal relaxation time (T_1_) was determined by 180°–90° inversion recovery pulse sequence. 16 values of delay time (τ) were applied and 16 scans for each τ value were recorded. The preacquisition delay (D1) was set to 2 × T_1_ (5 s) of the longest relaxation time. The value of the longitudinal relaxation time was obtained with the help of the T1/T2 relaxation module of Topspin as described in the manual of this software whereas the fitting function “invec” and fitting type “area” was used.

### Molecular docking studies

Schrödinger Release 2015-4 software was used for molecular docking studies.

#### Protein preparation

The crystal coordinates of COX-1 and COX-2 with PDB ID 1DIY and 1CVU, respectively were taken (protein data bank) and refined by using the protein preparation wizards of the software. Hydrogens, missing side chains and atoms were added and the bond orders were assigned to the two protein structures. Further refinement of the protein consists of ionization of the heteroatoms by epik^37,38^ at biological pH. H-Bonds were optimized for reducing the steric clashes by histidine, aspartate, glutamate and hydroxyl containing amino acids. OPLS 2005 force field was used to minimize the protein structure^39–41^.

#### Ligand Preparation

The ligprep tool of the software was used to convert 1D/2D structures to 3D and for the optimization of the ligand. The ligand geometry was energy minimized by using OPLS 2005 force field.

#### Molecular Docking

The molecular docking of the compounds in the active site of the enzyme was performed by using induced fit tool of the software. Taking the prepared ligands and the protein; Glide and Prime jobs were run simultaneously. Glide did the docking of ligand into the specified active site of receptor while prime continued the structure refinement of docked complex of ligand and enzyme. All the calculations were run in extra precision mode with standard sampling. Best docked pose of the enzyme - ligand complex was taken for interpretation.

## Electronic supplementary material

Supplementary material
